# Interobserver and intraobserver variability in the radiological assessment of sialolithiasis using cone beam computed tomography

**DOI:** 10.4317/medoral.24980

**Published:** 2021-10-27

**Authors:** Lotte J Beumer, Erik H van der Meij, Jolanda I Kamstra, Jan GAM de Visscher

**Affiliations:** 1 DDS, MD. Department of Oral and Maxillofacial Surgery, Medical Centre Leeuwarden, Leeuwarden, the Netherlands; 2 DDS, MD. Department of Oral and Maxillofacial Surgery, University Medical Center Groningen, the Netherlands; 3 DDS, MD, PhD. Department of Oral and Maxillofacial Surgery, Medical Centre Leeuwarden, the Netherlands; 4 DDS, MD, PhD. Department of Oral and Maxillofacial Surgery, University Medical Center Groningen, the Netherlands; 5 DDS, MD, PhD. Department of Oral and Maxillofacial Surgery and Oral Pathology, Amsterdam University Medical Center location VUMC/Academic Centre for Dentistry Amsterdam (ACTA), The Netherlands

## Abstract

**Background:**

Data regarding the inter- and intraobserver variability in the radiological assessment of sialolithiasis using cone beam computed tomography are missing in the current literature. This study assessed the inter- and intraobserver variability in the radiological assessment of sialolithiasis using cone beam computed tomography (CBCT).

**Material and Methods:**

In 107 patients, 130 salivary glands (65 parotid and 65 submandibular) with clinical signs of obstruction were assessed by four independent observers; 2 residents OMFS and 2 experienced OMFS. The observers analyzed the CBCT images and determined the absence or presence of one or more salivary stones in the affected gland. This procedure was repeated after three months.

**Results:**

Interobserver agreements showed kappa values of 0.84 for the parotid gland, and 0.93 for the submandibular gland. Intraobserver agreements for the whole group reported kappa values between 0.83 - 0.95. There was no significant difference between residents and experienced OMFS.

**Conclusions:**

Due to the good inter- and intraobserver agreement, CBCT appears to be a reproducible imaging modality for detecting salivary stones in patients with signs and symptoms of obstructed parotid and submandibular glands.

** Key words:**Salivary gland calculi, cone-beam computed tomography, observer variation.

## Introduction

Symptomatic sialolithiasis is a disease whereby the outflow of saliva of a major salivary gland is mechanically blocked due to calculi within the salivary duct. A salivary stone originates from a nidus that is composed of various ratios of organic and inorganic substances and can eventually lead to obstruction of a salivary gland duct. Patients often present with recurrent, sometimes mealtime-related, painful unilateral gland swelling. This can remain transitory or be complicated by a bacterial superinfection.

Postmortem studies indicate that salivary stones are present in 1.2% of the population (1). Literature estimates their annual symptomatic incidence at 1 per 10,000-30,000 individuals (2). Most salivary stones, about 80% to 90%, are localized in the submandibular gland and its duct (1,3,4). Marchal *et al* reported that parotid glands are affected up to 40% of the cases which is very high percentage and may be explained by the sensitivity of the detection methods used (2). Salivary stones are very rare in the sublingual and accessory salivary glands.

Multiple imaging modalities for diagnosing sialolithiasis, are used including two-dimensional (2D) radiography, ultrasonography (US), magnetic resonance imaging (MRI), computed tomography (CT) and sialography. Each of these imaging modalities has its own advantages and disadvantages. US and 2D radiography are routinely used owing to the readily availability cost-effectiveness, and absence and lower radiation dose, respectively (5).

Recently more research attention is being paid to the conebeam computed tomography (CBCT) application for salivary stone diagnosis. A mean sensitivity and specificity for salivary calculus diagnosis of 98,85% was reported in a retrospective study (5). Another retrospective study compared US and CBCT data in a cohort of 43 patients with clinical suspicion of sialolithiases (6). It was concluded that CBCT has a superior sensitivity compared to US but that the use of CBCT must be critically evaluated and should not be used as a primary option because of the radiation.

A prospective study to evaluate the value of CBCT in de detection of salivary stones prior to sialendoscopy, was performed by our research group (7). A sensitivity of 94%, a specificity of 90%, a positive predictive value of 84% and a negative predictive value of 97% was found. Based on these finding, we concluded that CBCT seems to be the ideal first-line imaging modality in patients with signs and symptoms of obstructed major salivary glands.

However, data regarding the inter- and intraobserver variability on the interpretation of CBCT images related to the possible presence of salivary stones are missing. But imaging techniques do not make diagnoses; rather, they aid observers who make the diagnosis (8-10). Observers possess different cognitive, visual, and perceptual abilities (10). To understand the performance of medical imaging technology, there is need to study the critical components of the technology, including the observers (10). The aim of this retrospective study was to assess the inter- and intraobserver variability in the diagnostic assessment of salivary calculi on CBCT-scans. This information is needed to decide whether to use CBCT as first choice imaging modality for patients with suspected sialolithiasis.

## Material and Methods

The patients were referred to the Department of Oral and Maxillofacial Surgery of the Medical Centre Leeuwarden, the Netherlands, for the diagnosis and management of salivary gland obstruction of the submandibular or parotid gland during the period January 2012 to August 2016. A CBCT scan was performed at the first visit to determine the absence or presence of one or more salivary stones. The selection of this cohort was described in our prospective study (7), 28 patients were excluded because of missing data. A total number of 130 affected glands (65 parotid and 65 submandibular) in the group of 107 patients (59 females and 48 males) were investigated.

All CBCT images were acquired with a PaX-Zenith 3D scanner (Vatech, Hwaseong, Republic of Korea) (FOV 16x16x12 cm, voxel size 0.2 mm). The CBCT images were retrospectively analyzed using a workstation, which was technically approved for radiological diagnostics, consisting of a 17-inch LCD monitor and a computer. Four observers participated in this study: two experienced oral and maxillofacial (OMF) surgeons with each more than twenty years of clinical experience and two residents of OMF surgery with each a minimum of 2 years clinical experience. All the observers interpret daily CBCT images. The observers had freedom to navigate the CBCT images and they could adjust the greyscale.

In the first session the observers were provided with the set of CBCT images and the specification of the affected gland (parotid or submandibular, left or right). No further clinical information or patient data were given to the observer. The reviewing observers were fully aware that their judgment would be compared to others and had no calibration exercises beforehand. The observers analyzed the CBCT images and determined the absence or presence of one or more salivary stones in the affected gland (no stone, or one stone or more stones). Three months after the first session, each of the four observers repeated the procedure. The order of data of the set was randomly changed.

- Statistical analysis

The collected data were analyzed using statistical software SPSS (IBM SPSS Statistics 26) and R (version 3.6.3 (r77832)). R was used for assessment of the interobserver variability of all observers by calculation of Lights Kappa and kappa statistics of SPSS was used for assessment of the intraobserver variability by calculation of Cohens Kappa (11). Kappa (κ) score is commonly used to evaluate reliability of paired agreements against pure chance agreement [range 0 (random agreement) to 1 (perfect agreement)] (12). The following grading of κ values was used: <0.20: poor agreement; 0.21-0.40 fair agreement; 0.41-0.60 moderate agreement; 0.61-0.80 substantial agreement; >0.80 good agreement (12).

## Results

Inter- and intraobserver variability rates are summarized in Table 1 and Table 2. Interobserver agreements defined by kappa was 0.84 for the parotid gland and 0.93 for the submandibular gland with an interobserver agreements for both salivary glands of 0.91 (Table 1). Intraobserver agreements for the parotid gland varied between 0.74 to 0.95, for the submandibular gland between 0.85 to 0.94 and for both salivary glands between 0.83 to 0.95. (Table 2). In Table 3 the sialendoscopic findings (the actual presence or absence of a sialolith) of our previous study (7) are summarized.

## Discussion

**Figure 1:**
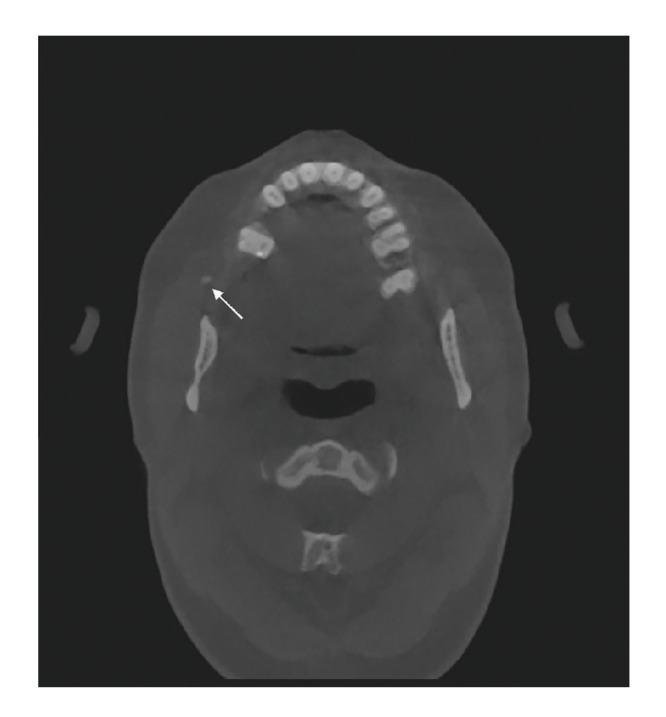
A round radiopacity (arrow) is located in the Stensens duct of the parotid gland.

**Figure 2:**
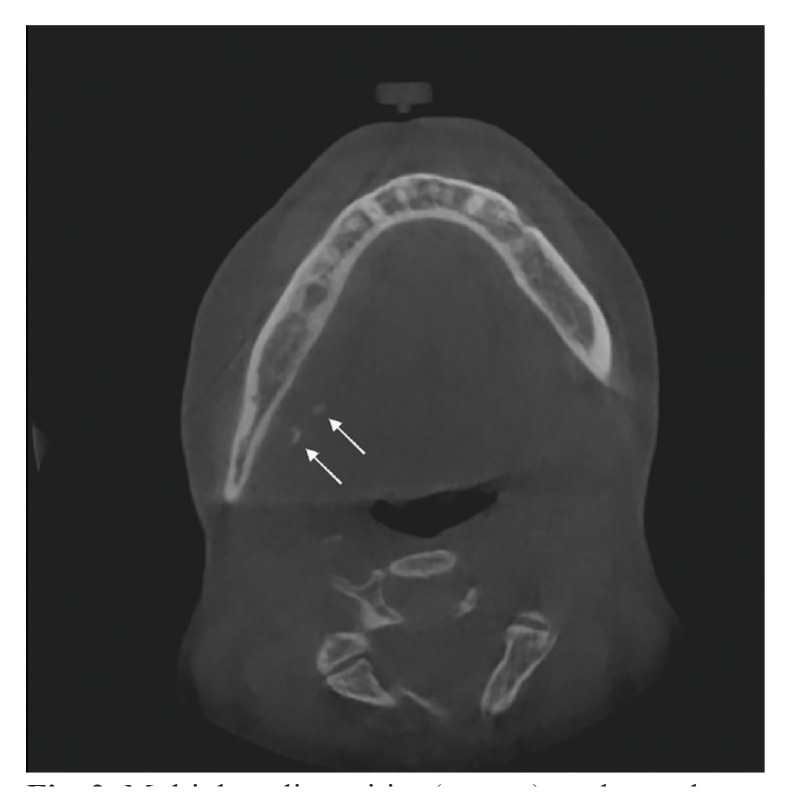
Multiple radiopacities (arrows) are located near the hilum of the submandibular gland.

The results of the present study demonstrate good agreement in the radiological assessment of the absence or presence of salivary stones in the parotid and submandibular gland (Fig. 1, Fig. 2) by using CBCT. The assessments were reproducible and not observer-dependent, indicating that the reviews of the four observers are comparable with each other. No differences in intraobserver variability in experienced OMF surgeons and in residents OMF surgery were found. One might expect that experienced OMF surgeons would do better as a result of their many years of experience.

**Table 1:**
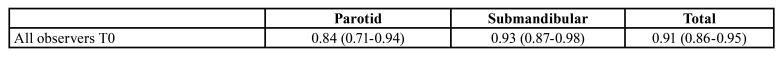
The Lights Kappa Coefﬁcient (Bootstrapped 90% CI (two-sided)) for interobserver variability of assessment of salivary stones using CBCT images (no stone versus stone(s)).

**Table 2:**
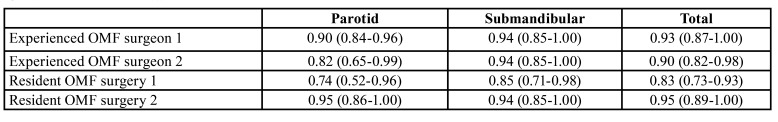
The mean Cohens Kappa Coefﬁcient (95% CI interval) for intraobserver variability of assessment of salivary stones using CBCT images (no stone versus stone(s)).

**Table 3:**
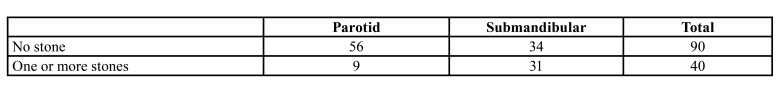
The sialendoscopic findings: presence or absence of a sialolith.

The result may be explained by the routinely daily exposure and therefore experience with interpretation of medical CT and CBCT images for various reasons by residents.

**Figure 3:**
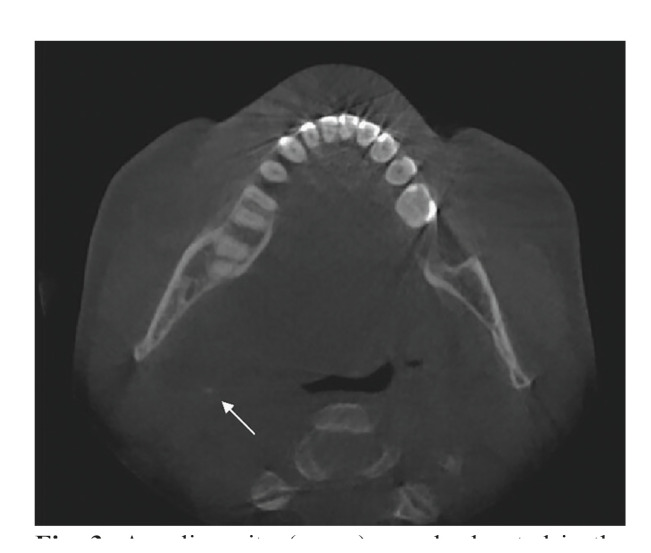
A radiopacity (arrow) may be located in the deep lobe of the parotid gland, the uncinate processs of the submandibular gland or in the palatine tonsil.

**Figure 4:**
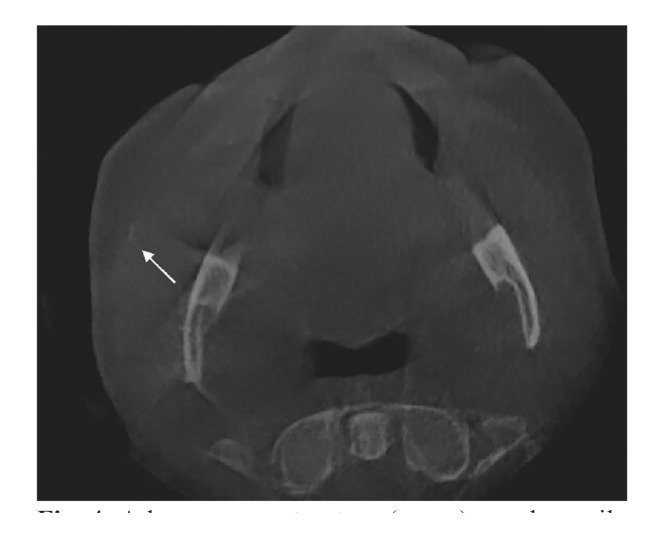
A less opaque structure (arrow) may be easily missed (proximal in the right parotid duct).

In this study, cases with inter- or intraobserver disagreement showed specific diagnostic difficulties. Firstly, some cases were difficult to interpret due to anatomic interpretations, especially in the area on the medial site of the ascending ramus of the mandible where it may be hard to distinguish between stones in the deep lobe of the parotid gland, the uncinate processs of the submandibular gland, or the tonsil (Fig. 3). Secondly, several cases are difficult due to the limited opacity of the salivary stones. Those stones can be easily missed while in addition the opacity is sometimes only seen in one slide of the CBCT (Fig. 4).

A limitation of the current study is the exclusion of 28 patients from the original cohort due to missing data. Another limitation is that the data do not indicate whether the observers agreed in their assessment on the same calcification. In other words, observations with a true positive and false positive outcome could be scored as agreement.

Other studies that have determined the inter- and intraobserver variability in the diagnostic assessment of various aspects on CBCT showed comparable good results, including buccal bone measurements of dental implants, assessment of temporomandibular joint condylar morphology, detection of periodontal defects, identification of apical periodontitis, assessment of impacted mandibular third molars, and mandibular condyle fractures (13-18). These studies showed a good reproducibility in observing mineralized tissues. In three-dimensional evaluating of the soft tissue of the oropharynx morphology, CBCT has proven to be a reliable diagnostic tool with good intraobserver agreement (19).

Besides CBCT, a variety of imaging modalities is used for the detection of salivary stones. Each of these modalities has its advantages and disadvantages such as the sensitivity and specificity for the diagnosis of a salivary stone, dose of ionizing radiation, the costs and the readily availability of the technique in daily practice.

US is the least invasive method. Based on low costs, high availability, and no radiation dosages, US is routinely used in many practices (5). The sensitivity of US is limited, with a reported sensitivity of 65% and a negative predictive value of 21% when using sialendoscopy as the golden standard (20). There is a great variability in ultrasonographer experience and comfort with salivary gland pathology (21). The interobserver agreement of six reviewers, who only assessed the ultrasound video recordings and were blinded to all other information, was found ranging from substantial to good agreement between observer pairs with a κ between 0.663 - 0.878 (22).

Due to the low costs, high availability and low radiation dosages, 2D radiography is often used in daily practice but the sensitivity is quite low with a reported rate of 60.7% (5). For intraobserver agreement, the kappa value was 0.52 for panoramic radiographs, and 0.64 for occlusal radiographs, indicating a minimum of moderate to substantial agreement. For interobserver agreement, the kappa value was 0.61 for panoramic radiographs, and 0.80 for occlusal radiographs, indicating at least substantial agreement (23).

Medical CT displays a high accuracy in the detection of salivary stones, with a reported sensitivity rate of 98% when using sialendoscopy as a gold standard (20). In our previous study, sensitivity and specificity rates of CBCT were found to be 94% and 90%, respectively, being comparable with those of a medical CT (7). Major advantages of CBCT above unenhanced CT are up to 15 times lower ionising radiation doses and its lower costs. Besides, CBCT is wider available as it is used routinely in most oral and maxillofacial units nowadays. Based on the data of our previous (7) and present study, CBCT seems to be a useful imaging modality with a high specificity and positive predictive value, and even higher sensitivity and negative predictive value and a good intra- and interobserver agreement.

When a stone is detected on a CBCT-scan in a patient with clinical signs and symptoms of salivary gland obstruction, the majority of cases require treatment. A variety of minimally invasive techniques is available for this purpose: sialendoscopy, transmucosal surgical approach, a combined approach and intra- or extracorporeal stone fragmentation.

Foletti *et al*. presented a therapeutic decision tree for determining the best minimally invasive technique to treat submandibular and parotid calculi, according to the diameter of the calculi and their position in the ductal system (24). They suggested to perform initial preclinical evaluation using a computed tomography (CT) scan or, rather, a CBCT scan in thin slices, possibly supplemented by Doppler ultrasonography (24). More research is needed to verify the position of CBCT in the treatment planning of sialolithiasis.

CBCT appears to be a reliable imaging modality for detecting salivary stones in patients with signs and symptoms of obstructed parotid and submandibular glands with a good intra- and interobserver agreement.
